# Differential rotational and translational movements along the thoraco-lumbo-sacral spine of load and non-load pulling military and police horses walking and trotting on hard and soft surfaces

**DOI:** 10.1186/s12917-026-05804-1

**Published:** 2026-09-08

**Authors:** K. Horan, S. E. Warner, P. Day, N. Housby-Skeggs, E. Lane-Ley, H. Telfer, D. Berner

**Affiliations:** 1 The Royal Veterinary College, Hawkshead Lane, Hatfield, Hertfordshire, AL9 7TA UK; 2 The Horse Trust, Slad Lane, Princes Risborough, Buckinghamshire, HP27 0PP UK; 3 The King’s Troop Royal Horse Artillery, King George VI Lines, Repository Road, Woolwich, Greater London, SE18 4BB UK; 4 Defence Animal Training Regiment, Remount Barracks, Asfordby Road, Melton Mowbray, Leicestershire, LE13 0HX UK

**Keywords:** Equine, Back, Range of motion, Rotational, Translational, Discipline, Surface

## Abstract

To improve detection of equine back-related disorders, it is important to establish how movement dynamics in horses’ thoraco-lumbo-sacral spine are normally affected by intrinsic and extrinsic factors. This cross-sectional study aimed to quantify how discipline, gait, and surface impact differential rotational and translational movements along the backs of 107 horses from the military and police forces. The horses were walked and trotted in-hand in a straight-line over hard and soft surfaces. During the exercises, nine inertial sensors (XSens MTw) were mounted to the poll, withers, thoracic vertebrae-13 (T13) and 18 (T18), lumbar vertebra-3 (L3), sacrum (between tubera sacrale), left and right tubera coxae, and caudal sacrum (‘tail’). Linear mixed models assessed whether horse discipline (load pulling or non-load pulling), surface (soft or hard), age, or stride time influenced rotational and translational range of motion differences between adjacent sensors at either walk or trot (significance at *p* < 0.05). When compared to non-load pullers, it was apparent that the load pullers had reductions in differential rotational movements (0.5–3.1º in walk, *p* ≤ 0.039; and 0.5–2.3º in trot, *p* ≤ 0.049) and reductions in differential cranio-caudal, medio-lateral and dorso-ventral translations (2.2–15.0 mm in walk, *p* ≤ 0.039; and 1.2–7.6 mm, *p* ≤ 0.045 in trot), plus an increase in differential cranio-caudal translation at the Poll-Withers (12.8 mm in trot, *p* < 0.001). Soft surfaces increased differential rotational movements (0.1–1.2º in walk, *p* ≤ 0.048; and 0.2–2.9º in trot, *p* ≤ 0.007) and differential translational movements (1.4–16.3 mm in walk, *p* ≤ 0.002; and 0.5–2 mm in trot, *p* ≤ 0.007) in most regions of the thoraco-lumbo-sacral spine compared to hard surfaces. The amplitude of differential rotational and translational movements did not show systematic patterns with stride time and age. Further work is needed to understand whether the observed changes to differential rotational and translational movements along the equine thoraco-lumbo-sacral spine, imposed by working discipline and surface conditions, impact long-term equine health, well-being, or susceptibility to injury. This work will lay foundations for the development of an equine back dynamics reference framework, to which individual data may be compared.

**Supplementary Information:**

The online version contains supplementary material available at https://doi.org/10.1186/s12917-026-05804-1.

## Introduction

Back problems are a significant health and welfare concern in horses. There is a need to establish technologies that quantify health, predict disease onset, aid disease diagnosis, and facilitate understanding of back disease aetiology. However, at present, dynamic evaluations of horses referred for back pathologies in clinical practice are mostly subjective and prone to bias with, typically, an unridden assessment first being used to try to rule out lameness, followed by a ridden assessment [9]. With the advent of mobile and practical assessment techniques, including inertial sensing and wireless data transmission [16, 61], our understanding about lameness and movement asymmetry in horses has expanded considerably [26, 45, 49]. For example, inertial measurement units (IMUs) have facilitated quantitative assessment of vertical upper body movement during clinically relevant exercises, such as flexion tests [36, 57, 58], circular movement on the lunge [44, 50] and after diagnostic analgesia [14, 33–35, 43, 53], thereby limiting the influence of possible sources of bias in the diagnostic process [1] and helping to overcome the limits of visual assessment [40]. These same practically relevant objective techniques are now also available to quantitatively assess back movement in horses. Subtle variations in back movements are particularly difficult to identify with the human eye [6] due to the stabilisation of the thoraco-lumbo-sacral area by hypaxial and epaxial musculature. This musculature limits rotation within most intervertebral joints to less than 5 degrees at trot [8]. Therefore, the recent advances in inertial sensing technology offer great potential to aid in the clinical assessment of back pathologies.

Nonetheless, to provide context for the interpretation of back movement abnormalities, it is necessary to first quantify normal back movement in the horse. It is also important to understand how intrinsic and extrinsic factors may impact movement, as these will inevitability coincide with any potential pathological issues and must be accounted for during gait assessments. Range of motion and differential movements between anatomical sites in the thoraco-lumbo-sacral region, are known to be altered by the presence of a rider [31, 32], ridden position [37] and saddle fit [29], as well as surface and shoeing conditions [4]. For example, the addition of weight (e.g. a rider) and a saddle induce an overall extension of the thoracolumbar spine [6], and hollowing of the thoracolumbar spine increases with the magnitude of the load [55]. It is to be expected that a horse’s age, breed, and discipline will also affect motion in the thoraco-lumbo-sacral spine. In addition, pathological conditions may impact back movement, such as lameness. Lameness is one of the most prevalent health problems in the horse and lameness and back disorders commonly co-exist: 74% of horses with back problems have lameness issues and 32% of lame horses have back problems [27]. It has been observed that the resolution of hindlimb lameness can increase the range of motion in the thoracolumbar region and reduce asymmetry [12].

The purpose of this study was to evaluate the impact of working discipline on equine back movement in healthy military/police horses free from any known back and lameness issues. Specifically, this work sought to investigate whether load versus non-load pulling activities would result in contrasting ranges of movement along the thoraco-lumbo-sacral spine using inertial sensing technology, considering both translational and rotational movements during locomotion on hard and soft surfaces. Any differences identified between the two groups of healthy horses, would suggest there is a longer-term impact of discipline on the mechanics of their movement, which is relevant for their long-term health and management. Prioritising good health in these horses is essential, with welfare, performance, and financial implications. A greater understanding of the impact of daily work on equine back health, will enable earlier diagnosis of issues, more timely treatment, tailored rehabilitation, earlier return to work, and fewer days lost to injury.

## Methods

### Ethics

Ethical approval for this study was received from the Royal Veterinary College Clinical Research Ethical Review Board (URN 2021 2025–3), which included a written consent form for horse owners.

### Horse participants

Horses were recruited from the Defence Animal Training Regiment (DATR) in Melton Mowbray, the Kings Troop Royal Horse Artillery in London, and The Horse Trust in Buckinghamshire, and were sub-divided into the following two discipline groups:

1. Military horses with load pulling specialism: 49 horses (42 active at The Kings Troop Royal Horse Artillery and 7 retired at the Horse Trust). To be included in the ‘load-pulling’ discipline, horses were either currently involved in (active subgroup) or had previously been involved in (retired subgroup) around 6 h per week of loading pulling training. The Kings Troop horses are required to pull 13-pounder guns, as part of gun carriage processions and state gun salutes. Six horses pull the guns, with the three on the right-hand side being ridden, and the horses alternate between being ridden and non-ridden when in a team. To prepare for these events, the Kings Troop horses participate in regular drill practice at the Royal Artillery Barracks in London (Woolwich). Typically, drill practice involves two load pulling training sessions per week, which are each 3 h in duration, and this includes time where the horses are static in harness, and horses may move in and out of the load pulling roles during sessions. During the load pulling exercises, the horses are either ridden or in-hand, and they spend approximately 50% of their training time in each condition. In addition to the 6 h of specialised load-pulling exercise, the Kings Troop horses complete four training sessions of 60 min duration without load pulling each week, and again this work is split between 50% ridden and 50% in-hand exercise. Two of the retired horses based at the Horse Trust previously served in the Kings Troop, while the other five had been retired from The Royal Mews (Table 1).
2. Non-load pulling horses: 58 horses (26 active horses at the DATR Military Barracks and 32 retired horses at the Horse Trust). To be included in the ‘non-load pulling’ discipline, horses were either currently involved in (active subgroup) or had previously been involved in (retired subgroup) ridden military/police activities devoid of any load-pulling exercise. The horses at DATR are used for rider training and complete five ridden sessions per week, of which 2–3 are jumping and 2–3 are flatwork. They also do one lunge session each week and will generally spend 30 min on a walker or have turnout daily. The (retired) Horse Trust horses included 20 police horses and 12 horses that had been ridden in the military (e.g. Light Cavalry) (Table 1).

**Table 1:** Key features of the two horse discipline groups. Mean estimated body mass was determined using trunk circumference data and a weigh tape

**Horse discipline**	**Working status**	**n**	**Load-pulling workload per week**	**Other workload per week**	**Mean age (months)** **(mean ± 2 S.D.)**	**Height (cm)** **(mean ± 2 S.D.)**	**BCS (scale 1–5)** **(mean ± 2 S.D.)**	**Trunk circumference (cm) (mean ± 2 S.D.)**	**Mean estimated body mass (kg)**
Load pulling	Active	42	6 h, ridden and in hand (2 sessions)	2 h of ridden exercise (2 sessions) 2 h of in-hand exercise (2 sessions)	148 ± 106	163 ± 9	Neck/shoulders: 3.1 ± 0.8 Trunk: 3.3 ± 0.9 Hindquarters: 3.0 ± 0.6	198 ± 14	546
	Retired	7	None	None					
Non-loading pulling	Active	26	None	Ridden exercise, jumping or flatwork (5 sessions) Lunging exercise (1 session)	216 ± 174	169 ± 10	Neck/shoulders: 2.8 ± 1.1 Trunk: 3.0 ± 0.9 Hindquarters: 2.8 ± 0.7	205 ± 14	596
	Retired	32	None	None					

The load pulling horses ranged in age from 76 to 324 months, with a mean age of 148 ± 106 months (mean ± 2 s.d., unless otherwise stated), and their heights ranged from 156 to 179 cm, with a mean height of 163 ± 9 cm. Body condition score was evaluated using a 1–5 point scale [3] and found to be 3.1 ± 0.8 at the neck/shoulders, 3.3 ± 0.9 at the trunk, and 3.0 ± 0.6 at the hindquarters in the load pulling horses. Trunk circumference (around T8) ranged from 186 to 228 cm, with a mean value of 198 ± 14 cm. The trunk circumference provides a corresponding estimate for body mass of 464 to 770 kg, and a mean of 546 kg on the Danilon weigh tape scale, although this may be a slight underestimate [15]. Non-load pulling horses ranged in age from 58 to 348 months, with a mean age of 216 ± 174 months, and their heights ranged from 157 to 178 cm, with a mean height of 169 ± 10 cm. Their body condition score was 2.8 ± 1.1 at the neck/shoulders, 3.0 ± 0.9 at the trunk, and 2.8 ± 0.7 at the hindquarters. Trunk circumference (around T8) in the non-load pullers ranged from 191 to 220 cm, with a mean value of 205 ± 14 cm. These values for trunk circumference correspond to a body mass range of 498 to 717 kg, and a mean of 596 kg on the Danilon weigh tape scale. Age data were unavailable for three non-load pulling horses, and height data and body condition scores were unavailable for two non-load pulling horses. Table 1 summarises the key features of the two horse groups.

Breeds varied but incorporated predominantly Irish Sports Horses. At The Kings Troop, there were 16 Irish Sports Horses, one Irish cross, one Cleveland Bay and one part-bred Cleveland Bay, one Irish Draft Cross, one Welsh part-bred, and 21 unknown breeds. At DATR, there were 22 Irish Sports Horses, two warmbloods, one KWPN and one Irish Draft horse. At the Horse Trust, there were 16 Irish Sports Horses, two Thoroughbred crosses, one Irish Draft and eight Irish Draft crosses, two Shire crosses, four warmbloods, one Welsh Section D and one Welsh cross, one cob cross and three Cleveland Bays. The retired horses had been out of work for between 6 months and 8 years, and the reasons for retirement were variable, including heart problems and osteoarthritis. Although an orthopaedic exam was not conducted prior to data collection, all horses were deemed sound by the veterinarian present at the time of data collection (N H–S, E L-L, or HT).

A sample size calculation based on dorsoventral and mediolateral ranges of motion in the mid-thoracic region – as the ‘centre of the back’, changes are expected to be most obvious in this region – between French Montagne stallions and a group of ‘general riding horses’ indicated required sample sizes between *N* = 8 and *N* = 28 per group (data from [17]). Within each discipline group, we therefore aimed to collect data from *N* > 30 horses.

### Equipment

**Fig. 1:**
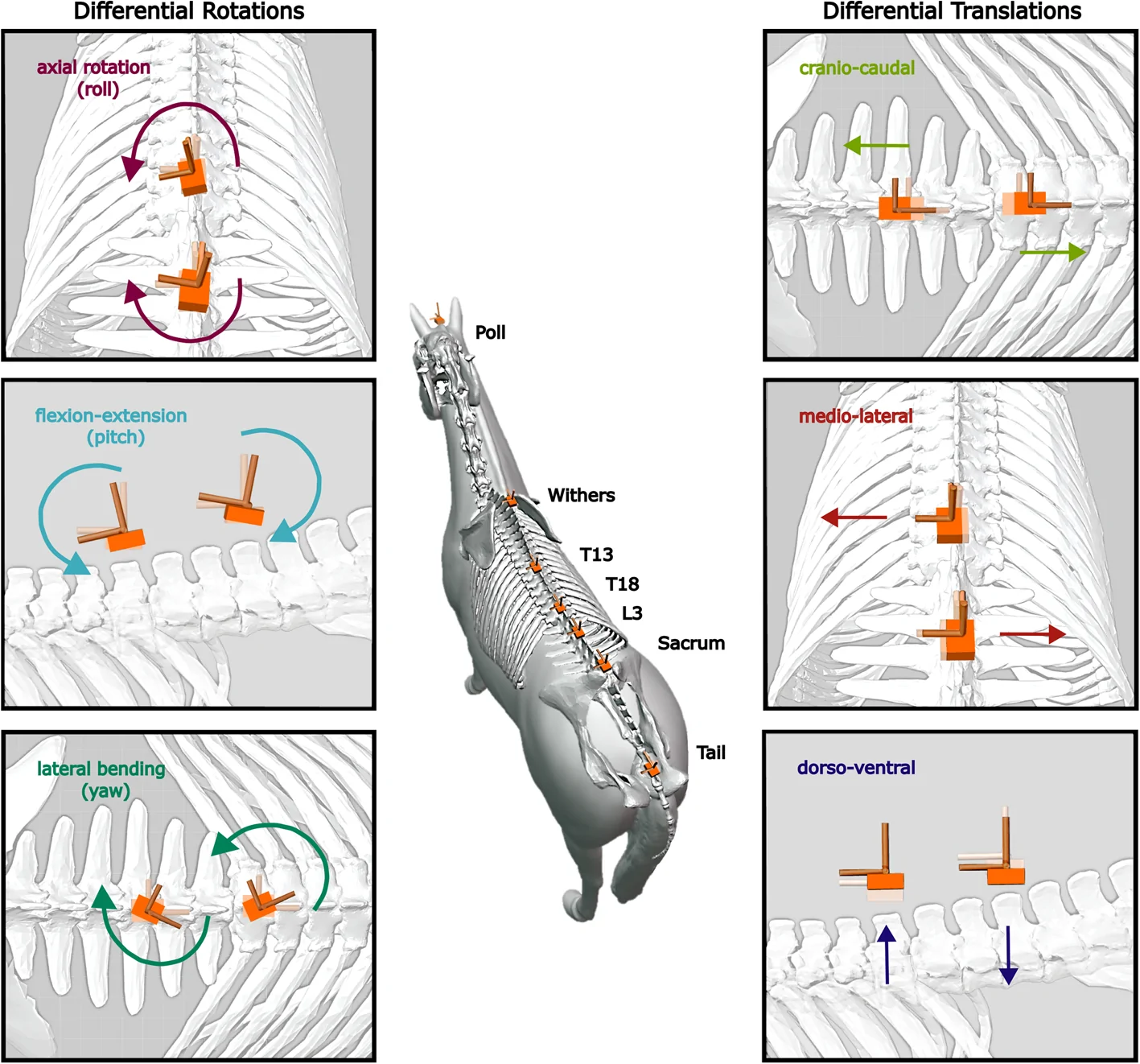
Position of sensors in relation to horse anatomy. Sensors were mounted non-invasively to each horse’s skin/hair with double-sided tape. The skeleton images were adapted from Rowaic [52]

Nine IMU devices (XSens MTw), each with a mass of 16 g, were positioned at the poll, withers, thoracic vertebrae 13 (T13), thoracic vertebrae 18 (T18), the third lumbar vertebra (L3), left and right tubera coxae, sacrum (between tubera sacrale) and the caudal sacrum of the horses, as part of a validated sensor-based system [47, 61] (Fig. 1). The same researcher (KH) identified the anatomical positions on all horses by palpation and then secured the devices using custom-made Velcro pouches and doubled-sided tape. IMU data were collected with an update rate of 60 Hz per individual sensor channel and transmitted to a receiver station (Awinda, Xsens) via a proprietary wireless data transmission protocol (XSens), which was connected to a laptop computer (Windows 11 Pro, 64-bit operating system, 16 GB RAM, Intel(R) Core (TM)i7-10610U CPU processor, 512 GB disk space) running MTManager (XSens) software.

Differential ranges of movement were calculated from the inertial sensor data between the following anatomical locations: Poll-Withers, Withers-T13, T13-T18, T18-L3, L3-Sacrum (sacrum here referring to the sacrum between tubera sacrali) and Sacrum-Tail (tail in this instance referring to the caudal sacrum). There were two categories:

1. Differential rotational ranges of movement in three dimensions for axial rotation (roll), flexion–extension (pitch), and lateral bending (yaw), which were quantified in degrees.
2. Differential translational ranges of movement in three dimensions for cranio-caudal, medio-lateral, and dorso-ventral displacement, which were quantified in millimetres.

### Trial conditions

Each study horse undertook in-hand straight-line walk and trot exercise on hard (e.g. concrete, tarmac) and soft (e.g. sandy arena) surfaces at their home location. Walk data were unavailable for two horses (in one case this was due to the horse’s behavior, and in the second case it was because the horses’ companion was too distressed when an attempt was made to move to the soft surface) and trot data were unavailable for three horses (the two cases described above, plus one other instance of non-compliance in bad weather, including rearing under windy conditions). There were 47.6 ± 35.3 (mean ± 2 s.d.) strides per trial for the walk data, where stride time was 1225 ± 193 ms, and 36.5 ± 22.1 strides per trial for the trot data, where stride time was 728 ± 89 ms.

There were five soft surfaces and five hard surfaces used in this study. Mechanical surface properties were measured with a torque reader (both surface types), a moisture and temperature probe (soft surfaces only), and a Vienna Surface Tester (VST) (soft surfaces only). The VST is a mobile device consisting of a bowling ball instrumented with a global positioning system (GPS) and built-in accelerometers [11, 42, 46]. It is designed to be dropped onto a soft surface from increasing heights, from approximately 50 mm to 1000 mm, and it gathers data for impact velocity (in m/s) and impact acceleration (in multiples of gravitational acceleration), penetration depth (in mm), a coefficient of restitution that quantifies energy transferred from the surface back to the bowling ball during rebound (in %), and surface stiffness (in kN/m). Full quantitative details on the nature of the surfaces on different data collection days are provided in the supplement (Table S1). In some cases, surface properties were quantified more than once per day if a visual change in the conditions was observed, for example due to usage (associated with more or less compaction) or weather (e.g. wet or dry). In summary, average torque was 19 Nm for the hard surfaces and 14 Nm for the soft surfaces. The soft surfaces were characterised by an average moisture of 3.4% and an average temperature of 13.2ºC. Using linear regression analyses, average values for impact acceleration, penetration depth, energy restitution and stiffness were calculated to be 16.9 G, 16.5 mm, 2.1% and 172 kN/m, respectively, for an impact velocity of 2 m/s on the soft surface; the same parameters were 86.1 G, 24.3 mm, 2.1% and 310 kN/m, respectively, for an impact velocity of 4 m/s.

### Data processing and statistics

The IMU data were processed following published protocols [47]. In brief, the tri-axial sensor acceleration data were rotated into a gravity (z: vertical, positive up) and horse-based (x: cranio-caudal, positive forwards; and y: medio-lateral, positive to the left) right-handed reference frame and numerically double integrated to displacement. Displacement data were then segmented into individual strides based on the vertical velocity of the sacrum sensor [57, 58]. The data for each stride were linearly interpolated to 100-time percentage points and the average displacement across strides within a trial was calculated at each time point for each of the x, y, z axes of each sensor. Differential translational and rotational movements were calculated on a stride-by-stride basis from individual sensor displacement (x, y, z) and orientation data (roll, pitch, yaw) by subtracting the respective 100-time point signals from each other. This resulted in 100-time points of differential craniocaudal (x), mediolateral (y) and dorsoventral (z) translations as well as differential axial rotation, flexion–extension and lateral bending motions for each stride cycle. Differential range of motion (ROM) was calculated as the difference between the minimum and maximum value found for each stride cycle for each differential movement. Average differential ROM values across strides within trials were calculated.

Linear mixed models were implemented in IBM SPSS statistical software (version 28) to assess whether discipline or surface significantly affected the differential rotational and translational ROM between adjacent sensor positions. Horse was included as a random factor in the models, as we were not interested in the effects of individual horses. Stride time was included as a covariate (as well as a fixed factor) because we wanted to evaluate its numerical relationship with the outcome parameters; for example, stride time can be related to speed [41, 64] and it is important to establish whether the investigated variables increase or decrease with speed. We were unable to statistically compare active and retired horses due to the small sample size of the retired load-pulling subgroup (*n* = 7). However, age was included as a covariate (and a fixed factor) in the LMMs, as it is a well-defined characteristic that could be quantified and accounted for across all individuals. Nonetheless, we note that the age distributions of the horses from the two disciplines had only a limited amount of overlap. This means the model adjustments for age will be relying on some extrapolation beyond the limits of the observed data, and the effect of discipline is difficult to assess independently from the effect of age. For this reason, we repeated all 144 model runs without the age covariate and the outcomes of this analysis are compared to the original model runs (that included the age covariate, and which are presented below) in the supplement (Table S2). Age is a relevant covariate because older, retired horses may have a generally ‘stiffer’ gait, and this could be a confounding factor that might otherwise mask surface or discipline effects. Separate models were run for walk and trot due to previously reported fundamental differences between the two gaits [7, 8]. In all cases, models adopted the Restricted Maximum Likelihood method and the Satterthwaite approximation for the degrees of freedom. The significance threshold was set at *p* < 0.05. Histograms of model residuals were plotted and visually inspected for normality; no data transformations were deemed necessary. For each model, all post-hoc pairwise comparisons included Bonferroni correction.

## Results

### Overview

**Fig. 2:**
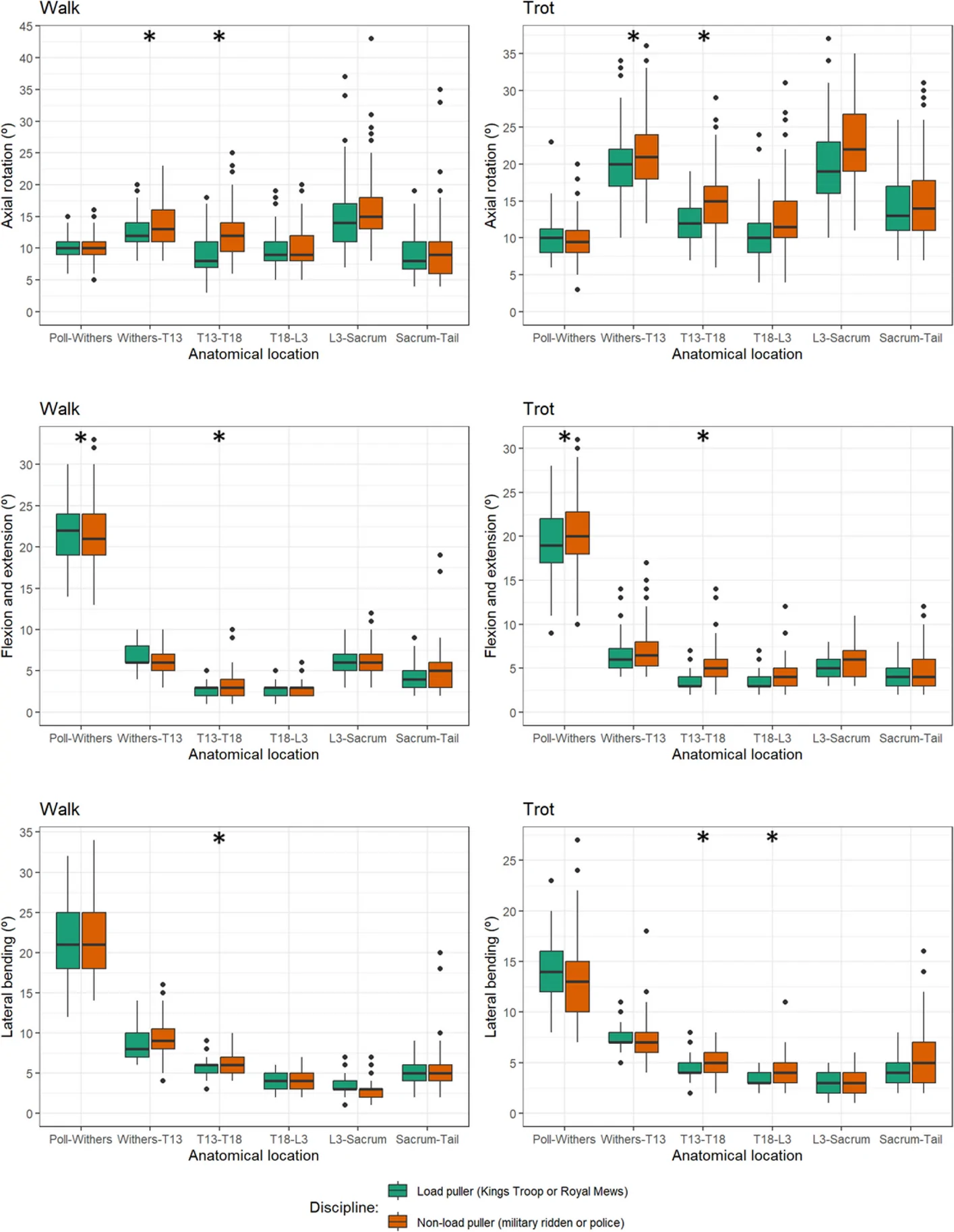
Influence of discipline on differential rotational back movements in military horses (*n* = 107). Anatomical regions found to be significantly affected by discipline in the linear mixed models are marked (*)

**Fig. 3:**
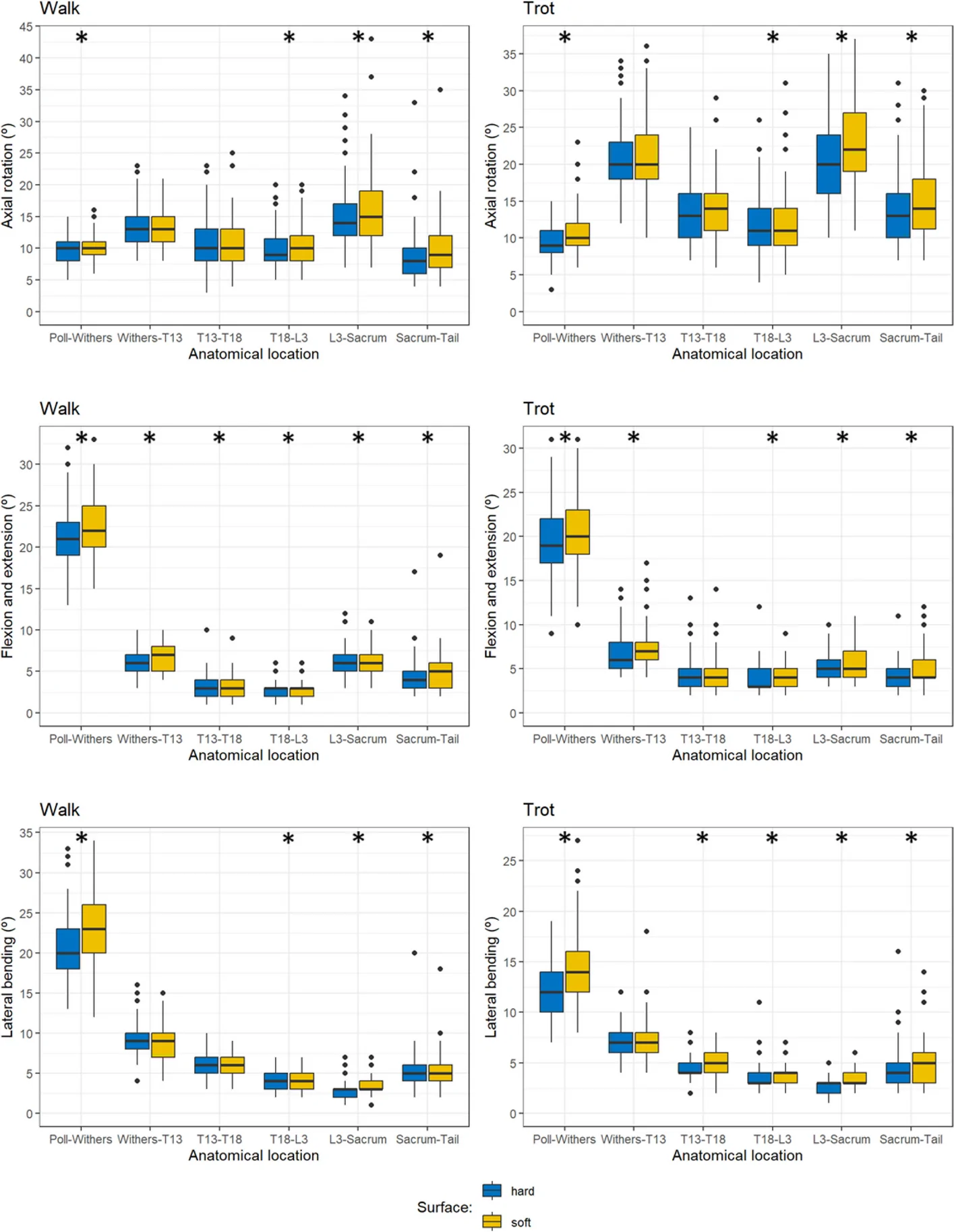
Influence of surface on differential rotational back movements in military horses (*n* = 107). Anatomical regions found to be significantly affected by surface in the linear mixed models are marked (*)

**Fig. 4:**
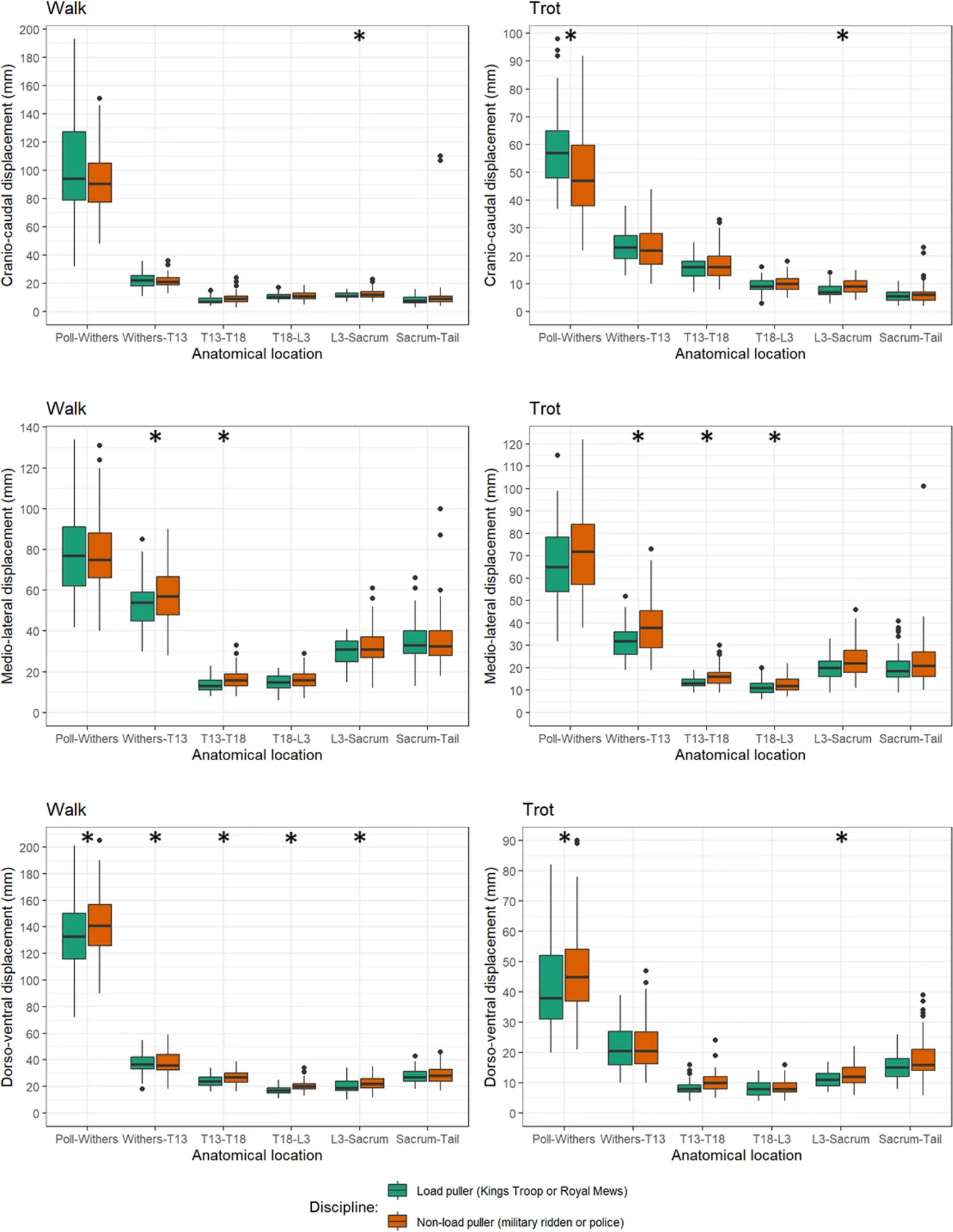
Influence of discipline on differential translational back movements in military horses (*n* = 107). Anatomical regions found to be significantly affected by discipline in the linear mixed models are marked (*)

**Fig. 5:**
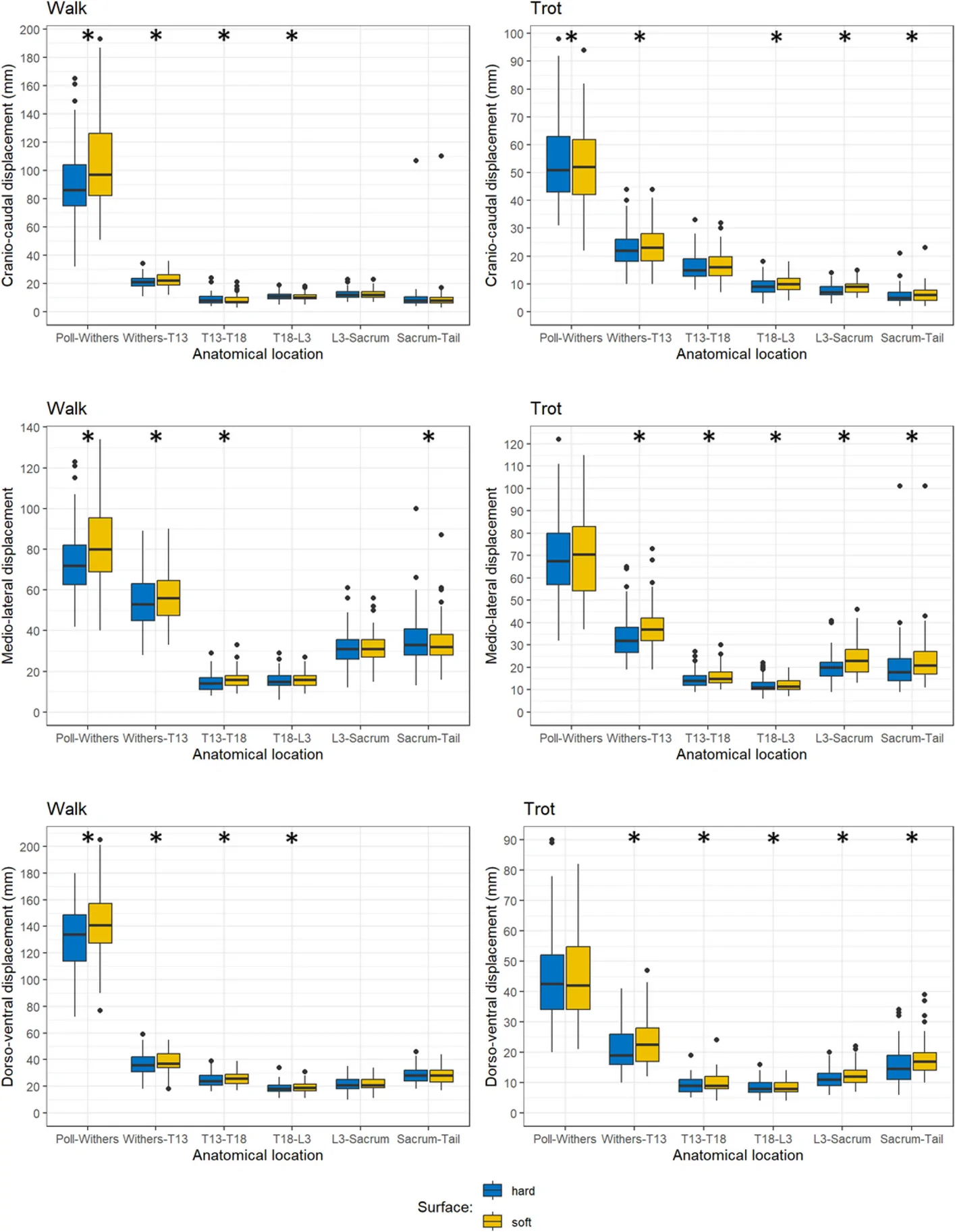
Influence of surface on differential translational back movements in military horses (*n* = 107). Anatomical regions found to be significantly affected by surface in the linear mixed models are marked (*)

The effects of the fixed factors (discipline and surface) on the differential and translational movements are reported below. Table 2 summarises the significance values from the linear mixed model outputs, and Tables 3 and 4 summarise estimated marginal means for discipline and surface effects, respectively. Figures 2 and 3 summarise the impact of discipline and surface, respectively, on differential rotational movements in the thoraco-lumbo-sacral spine. Figures 4 and 5 summarise the impact of discipline and surface, respectively, on differential translational movements in the thoraco-lumbo-sacral spine. In addition, we present data from a sub-set of the horses (active only) in Figures S1–S4, for visual comparison to the full horse dataset.

**Table 2:** *P*-values from linear mixed models. Values in bold reflect significance (*p* < 0.05)

**Anatomical Location**	**Factor**	**Differential axial rotation (roll)**		**Differential flexion and extension (pitch)**		**Differential lateral bending (yaw)**		**Differential cranio-caudal translation**		**Differential medio-lateral translation**		**Differential dorso-ventral translation**	
		**Walk**	**Trot**	**Walk**	**Trot**	**Walk**	**Trot**	**Walk**	**Trot**	**Walk**	**Trot**	**Walk**	**Trot**
		***P* value**	***P* value**	***P* value**	***P* value**	***P* value**	***P* value**	***P* value**	***P* value**	***P* value**	***P* value**	***P* value**	***P* value**
Poll-Withers	Discipline	0.058	0.976	**0.032**	**0.038**	0.501	0.602	0.604	**0.001**	0.453	0.205	**0.002**	**0.014**
	Surface	**0.001**	**< 0.001**	**< 0.001**	**0.001**	**< 0.001**	**< 0.001**	**< 0.001**	**0.020**	**< 0.001**	0.658	**< 0.001**	0.341
	Stride time	0.177	0.975	0.120	**0.021**	**0.001**	0.076	**< 0.001**	**0.001**	**0.004**	**0.001**	**0.001**	0.868
	Age	0.088	0.157	**0.001**	**0.001**	0.082	0.126	0.928	0.635	0.756	0.492	0.483	0.146
Withers-T13	Discipline	**0.009**	**0.046**	0.956	0.144	0.589	0.221	0.719	0.061	**0.001**	**0.001**	**0.038**	0.933
	Surface	0.064	0.396	**< 0.001**	**0.001**	0.681	0.421	**< 0.001**	**0.007**	**< 0.001**	**< 0.001**	**0.002**	**0.001**
	Stride time	0.137	0.064	**< 0.001**	0.934	0.176	0.527	**0.001**	**0.018**	**0.004**	**0.001**	0.132	**< 0.001**
	Age	0.882	0.099	0.740	0.855	**0.001**	0.196	0.065	**0.001**	**0.002**	**0.012**	**0.001**	**0.037**
T13-T18	Discipline	**0.001**	**0.004**	**0.001**	**< 0.001**	**0.039**	**0.026**	0.073	0.195	**0.001**	**0.001**	**< 0.001**	0.173
	Surface	0.679	0.111	**0.043**	0.089	0.307	**0.001**	**0.001**	0.097	**< 0.001**	**0.001**	**0.001**	**0.001**
	Stride time	0.053	0.161	**0.001**	**0.001**	0.779	**0.045**	0.735	**0.001**	**0.004**	**0.001**	**0.035**	0.128
	Age	**0.004**	0.601	0.064	0.240	0.667	0.300	0.419	0.985	0.175	**0.004**	**< 0.001**	**0.024**
T18-L3	Discipline	0.767	0.723	0.065	0.664	0.283	**0.049**	0.369	0.123	0.845	**0.045**	**0.001**	0.442
	Surface	**0.048**	**0.001**	**0.044**	**0.004**	**0.038**	**0.007**	**0.023**	**< 0.001**	0.099	**0.005**	**0.001**	**< 0.001**
	Stride time	**0.001**	0.517	**0.002**	0.961	0.505	0.424	0.260	0.150	**0.001**	0.299	0.131	**0.001**
	Age	**0.001**	**< 0.001**	0.108	**0.003**	0.408	0.670	0.142	0.077	**0.017**	0.429	0.068	**0.006**
L3-Sacrum	Discipline	0.175	0.164	0.878	0.718	0.935	0.725	**0.001**	**0.017**	0.856	0.387	**0.003**	**0.015**
	Surface	**< 0.001**	**< 0.001**	**0.025**	**< 0.001**	**< 0.001**	**< 0.001**	0.295	**< 0.001**	0.340	**< 0.001**	0.333	**0.001**
	Stride time	**< 0.001**	0.930	0.769	0.255	0.553	0.118	0.071	**0.022**	**< 0.001**	**0.006**	0.060	0.205
	Age	**0.001**	**0.001**	0.059	**0.001**	0.143	**0.002**	**0.003**	0.316	0.361	**0.003**	**0.006**	0.370
Sacrum-Tail	Discipline	0.460	0.639	0.089	0.353	0.911	0.715	0.111	0.286	0.742	0.496	0.843	0.266
	Surface	**< 0.001**	**< 0.001**	**< 0.001**	**< 0.001**	**0.001**	**< 0.001**	0.290	**< 0.001**	**0.001**	**< 0.001**	0.088	**< 0.001**
	Stride time	**0.006**	0.254	**0.021**	0.923	**0.022**	0.965	0.536	0.117	**0.015**	**0.009**	**< 0.001**	**0.015**
	Age	**0.046**	**0.023**	0.852	0.194	**0.004**	**< 0.001**	0.488	0.904	0.699	0.248	**0.037**	0.311

**Table 3:** Estimated marginal means for discipline effects

**Movement**	**Anatomical location**	**Discipline**	**Walk**				**Trot**			
			**Mean**	**Std. error**	**95% Confidence interval**		**Mean**	**Std. error**	**95% Confidence interval**	
					**Lower bound**	**Upper Bound**			**Lower bound**	**Upper bound**
Differential axial rotation (roll)	Poll-Withers	Load puller	9.6	0.3	9.0	10.1	10.0	0.4	9.3	10.8
		Non-load puller	10.3	0.3	9.8	10.8	10.0	0.3	9.3	10.7
	Withers-T13	Load puller	12.4	0.5	11.5	13.3	19.5	0.7	18.1	21.0
		Non-load puller	14.1	0.4	13.3	15.0	21.7	0.7	20.3	23.0
	T13-T18	Load puller	9.1	0.5	8.2	10.1	12.5	0.6	11.4	13.6
		Non-load puller	12.2	0.5	11.3	13.1	14.8	0.5	13.8	15.8
	T18-L3	Load puller	9.9	0.4	9.1	10.8	11.7	0.6	10.5	12.8
		Non-load puller	10.1	0.4	9.3	10.9	12.0	0.5	10.9	13.0
	L3-Sacrum	Load puller	14.8	0.7	13.3	16.2	20.9	0.8	19.4	22.5
		Non-load puller	16.2	0.7	14.8	17.5	22.5	0.7	21.1	23.9
	Sacrum-Tail	Load puller	8.8	0.6	7.7	10.0	14.4	0.7	13.0	15.9
		Non-load puller	9.5	0.6	8.4	10.6	14.9	0.7	13.6	16.3
Differential flexion extension (pitch)	Poll-Withers	Load puller	21.0	0.5	20.0	22.0	18.9	0.5	17.8	19.9
		Non-load puller	22.6	0.5	21.7	23.6	20.4	0.5	19.5	21.4
	Withers-T13	Load puller	6.4	0.2	5.9	6.8	6.5	0.4	5.8	7.2
		Non-load puller	6.3	0.2	6.0	6.7	7.2	0.3	6.6	7.9
	T13-T18	Load puller	2.5	0.2	2.1	2.8	3.7	0.3	3.2	4.2
		Non-load puller	3.6	0.2	3.3	3.9	5.4	0.2	5.0	5.9
	T18-L3	Load puller	2.7	0.1	2.4	3.0	3.9	0.2	3.4	4.3
		Non-load puller	3.1	0.1	2.8	3.3	4.0	0.2	3.6	4.4
	L3-Sacrum	Load puller	6.5	0.2	6.0	7.0	5.4	0.2	4.9	5.8
		Non-load puller	6.6	0.2	6.1	7.0	5.5	0.2	5.1	5.9
	Sacrum-Tail	Load puller	4.1	0.3	3.6	4.7	4.2	0.3	3.7	4.7
		Non-load puller	4.8	0.3	4.3	5.4	4.6	0.2	4.1	5.1
Differential lateral bending (yaw)	Poll-Withers	Load puller	21.5	0.6	20.2	22.8	13.6	0.4	12.7	14.5
		Non-load puller	22.2	0.6	21.0	23.3	13.3	0.4	12.5	14.1
	Withers-T13	Load puller	9.2	0.3	8.5	9.8	7.5	0.3	7.0	8.0
		Non-load puller	8.9	0.3	8.3	9.5	7.0	0.2	6.6	7.5
	T13-T18	Load puller	5.9	0.2	5.5	6.2	4.3	0.2	4.0	4.7
		Non-load puller	6.4	0.2	6.1	6.7	4.9	0.2	4.6	5.2
	T18-L3	Load puller	3.9	0.1	3.6	4.2	3.4	0.2	3.0	3.8
		Non-load puller	4.2	0.1	3.9	4.5	3.9	0.2	3.6	4.3
	L3-Sacrum	Load puller	3.1	0.2	2.8	3.5	3.1	0.1	2.9	3.4
		Non-load puller	3.1	0.1	2.8	3.4	3.2	0.1	3.0	3.5
	Sacrum-Tail	Load puller	5.3	0.3	4.7	5.9	4.7	0.3	4.1	5.3
		Non-load puller	5.2	0.3	4.7	5.8	4.8	0.3	4.3	5.4
Differential cranio-caudal translation	Poll-Withers	Load puller	99.3	4.2	91.1	107.6	59.7	2.0	55.7	63.7
		Non-load puller	96.2	3.9	88.4	103.9	46.9	1.8	43.3	50.6
	Withers-T13	Load puller	22.1	0.7	20.8	23.5	24.9	1.1	22.8	27.1
		Non-load puller	21.8	0.6	20.5	23.0	22.0	1.0	20.1	23.9
	T13-T18	Load puller	7.9	0.5	7.0	8.8	15.7	0.7	14.3	17.1
		Non-load puller	9.1	0.4	8.3	10.0	17.0	0.6	15.7	18.3
	T18-L3	Load puller	10.4	0.4	9.6	11.2	9.1	0.4	8.3	10.0
		Non-load puller	10.9	0.4	10.2	11.6	10.1	0.4	9.3	10.9
	L3-Sacrum	Load puller	10.8	0.4	10.0	11.6	7.7	0.3	7.1	8.4
		Non-load puller	13.0	0.4	12.2	13.7	8.9	0.3	8.3	9.6
	Sacrum-Tail	Load puller	7.5	1.5	4.5	10.6	5.6	0.4	4.8	6.5
		Non-load puller	11.1	1.4	8.2	14.0	6.3	0.4	5.5	7.1
Differential medio-lateral translation	Poll-Withers	Load puller	77.1	2.5	72.1	82.1	66.8	2.6	61.7	71.9
		Non-load puller	79.9	2.4	75.2	84.5	71.6	2.3	66.9	76.2
	Withers-T13	Load puller	50.1	1.7	46.7	53.5	31.4	1.4	28.7	34.1
		Non-load puller	60.4	1.6	57.2	63.6	39.0	1.2	36.5	41.4
	T13-T18	Load puller	13.2	0.6	12.0	14.3	13.4	0.5	12.3	14.4
		Non-load puller	17.0	0.5	16.0	18.1	16.5	0.5	15.6	17.5
	T18-L3	Load puller	15.7	0.5	14.6	16.7	11.3	0.5	10.3	12.2
		Non-load puller	15.8	0.5	14.8	16.8	12.7	0.5	11.8	13.6
	L3-Sacrum	Load puller	31.0	1.0	29.0	33.1	21.2	0.9	19.5	22.9
		Non-load puller	31.3	1.0	29.4	33.2	22.2	0.8	20.7	23.8
	Sacrum-Tail	Load puller	35.6	1.6	32.3	38.8	21.0	1.6	17.7	24.2
		Non-load puller	34.8	1.5	31.7	37.8	22.6	1.5	19.6	25.5
Differential dorso-ventral translation	Poll-Withers	Load puller	130.0	3.3	123.5	136.4	40.3	2.1	36.2	44.4
		Non-load puller	145.0	3.0	138.9	151.0	47.7	1.9	44.0	51.4
	Withers-T13	Load puller	36.0	1.1	33.7	38.2	21.7	1.0	19.8	23.6
		Non-load puller	39.5	1.1	37.3	41.6	21.6	0.9	19.8	23.3
	T13-T18	Load puller	22.5	0.7	21.2	23.8	8.9	0.4	8.1	9.7
		Non-load puller	28.1	0.6	26.9	29.4	9.7	0.4	9.0	10.4
	T18-L3	Load puller	16.7	0.5	15.6	17.7	8.2	0.4	7.4	8.9
		Non-load puller	20.6	0.5	19.6	21.6	8.6	0.3	7.9	9.2
	L3-Sacrum	Load puller	20.1	0.7	18.7	21.5	11.1	0.4	10.3	12.0
		Non-load puller	23.2	0.6	21.9	24.5	12.7	0.4	11.9	13.4
	Sacrum-Tail	Load puller	28.2	0.8	26.6	29.8	16.0	0.8	14.4	17.7
		Non-load puller	28.4	0.8	26.9	30.0	17.3	0.7	15.9	18.8

**Table 4:** Estimated marginal means for surface effects

**Movement**	**Anatomical location**	**Surface**	**Walk**				**Trot**			
			**Mean**	**Std. error**	**95% Confidence interval**		**Mean**	**Std. error**	**95% Confidence interval**	
					**Lower bound**	**Upper bound**			**Lower bound**	**Upper bound**
Differential axial rotation (roll)	Poll-Withers	Hard	9.7	0.2	9.3	10.0	9.4	0.3	8.9	10.0
		Soft	10.2	0.2	9.8	10.6	10.5	0.3	10.0	11.1
	Withers-T13	Hard	13.4	0.3	12.8	14.0	20.5	0.5	19.5	21.4
		Soft	13.0	0.3	12.4	13.6	20.7	0.5	19.8	21.7
	T13-T18	Hard	10.7	0.3	10.1	11.3	13.5	0.4	12.8	14.2
		Soft	10.6	0.3	10.0	11.3	13.8	0.4	13.1	14.5
	T18-L3	Hard	9.8	0.3	9.3	10.4	11.5	0.4	10.7	12.2
		Soft	10.2	0.3	9.6	10.7	12.2	0.4	11.4	12.9
	L3-Sacrum	Hard	14.9	0.5	13.9	15.8	20.4	0.5	19.3	21.4
		Soft	16.1	0.5	15.1	17.0	23.1	0.5	22.1	24.1
	Sacrum-Tail	Hard	8.6	0.4	7.8	9.4	13.7	0.5	12.8	14.7
		Soft	9.7	0.4	9.0	10.5	15.6	0.5	14.7	16.6
Differential flexion extension (pitch)	Poll-Withers	Hard	21.2	0.3	20.5	21.8	19.1	0.3	18.4	19.8
		Soft	22.5	0.3	21.8	23.2	20.2	0.3	19.5	20.9
	Withers-T13	Hard	6.0	0.1	5.7	6.3	6.6	0.2	6.1	7.0
		Soft	6.7	0.1	6.5	7.0	7.1	0.2	6.7	7.6
	T13-T18	Hard	3.1	0.1	2.9	3.3	4.5	0.2	4.1	4.8
		Soft	3.0	0.1	2.7	3.2	4.6	0.2	4.3	5.0
	T18-L3	Hard	2.8	0.1	2.6	3.0	3.8	0.1	3.5	4.1
		Soft	2.9	0.1	2.7	3.1	4.1	0.1	3.8	4.4
	L3-Sacrum	Hard	6.4	0.2	6.1	6.8	5.2	0.2	4.9	5.5
		Soft	6.6	0.2	6.3	7.0	5.7	0.2	5.4	6.0
	Sacrum-Tail	Hard	4.2	0.2	3.9	4.6	4.1	0.2	3.8	4.4
		Soft	4.7	0.2	4.4	5.1	4.7	0.2	4.4	5.0
Differential lateral bending (yaw)	Poll-Withers	Hard	20.4	0.4	19.5	21.2	12.2	0.3	11.6	12.8
		Soft	23.3	0.4	22.4	24.2	14.6	0.3	14.0	15.2
	Withers-T13	Hard	9.1	0.2	8.7	9.5	7.2	0.2	6.8	7.5
		Soft	9.0	0.2	8.6	9.4	7.3	0.2	7.0	7.6
	T13-T18	Hard	6.2	0.1	5.9	6.4	4.4	0.1	4.2	4.7
		Soft	6.1	0.1	5.9	6.3	4.8	0.1	4.6	5.0
	T18-L3	Hard	4.1	0.1	3.9	4.3	3.5	0.1	3.3	3.8
		Soft	4.0	0.1	3.8	4.2	3.8	0.1	3.6	4.1
	L3-Sacrum	Hard	2.9	0.1	2.7	3.1	3.0	0.1	2.8	3.1
		Soft	3.3	0.1	3.1	3.6	3.4	0.1	3.2	3.6
	Sacrum-Tail	Hard	5.1	0.2	4.7	5.5	4.5	0.2	4.1	4.9
		Soft	5.5	0.2	5.1	5.9	5.0	0.2	4.6	5.4
Differential cranio-caudal translation	Poll-Withers	Hard	89.6	2.8	84.0	95.2	54.4	1.3	51.8	57.1
		Soft	105.9	2.8	100.3	111.5	52.2	1.3	49.6	54.9
	Withers-T13	Hard	20.7	0.5	19.8	21.6	23.0	0.7	21.6	24.4
		Soft	23.2	0.5	22.3	24.1	24.0	0.7	22.6	25.4
	T13-T18	Hard	8.9	0.3	8.3	9.5	16.2	0.5	15.2	17.1
		Soft	8.2	0.3	7.6	8.8	16.5	0.5	15.6	17.4
	T18-L3	Hard	10.8	0.3	10.3	11.3	9.2	0.3	8.6	9.8
		Soft	10.5	0.3	10.0	11.0	10.0	0.3	9.5	10.6
	L3-Sacrum	Hard	11.9	0.3	11.4	12.5	7.8	0.2	7.3	8.2
		Soft	11.8	0.3	11.3	12.3	8.9	0.2	8.5	9.3
	Sacrum-Tail	Hard	9.2	1.0	7.2	11.2	5.7	0.3	5.1	6.2
		Soft	9.4	1.0	7.4	11.4	6.2	0.3	5.7	6.8
Differential medio-lateral translation	Poll-Withers	Hard	73.5	1.8	70.0	77.0	68.9	1.7	65.5	72.3
		Soft	83.5	1.8	80.0	87.0	69.5	1.7	66.1	72.9
	Withers-T13	Hard	53.5	1.1	51.2	55.7	33.0	0.9	31.3	34.8
		Soft	57.1	1.1	54.8	59.3	37.3	0.9	35.6	39.1
	T13-T18	Hard	14.4	0.4	13.6	15.2	14.6	0.3	13.9	15.3
		Soft	15.8	0.4	15.0	16.6	15.3	0.3	14.6	16.0
	T18-L3	Hard	15.9	0.4	15.2	16.6	11.7	0.3	11.1	12.4
		Soft	15.5	0.4	14.8	16.3	12.2	0.3	11.6	12.9
	L3-Sacrum	Hard	31.3	0.7	30.0	32.7	20.2	0.6	19.1	21.3
		Soft	31.0	0.7	29.7	32.4	23.2	0.6	22.1	24.3
	Sacrum-Tail	Hard	36.3	1.1	34.1	38.4	20.3	1.1	18.2	22.4
		Soft	34.1	1.1	31.9	36.2	23.2	1.1	21.2	25.3
Differential dorso-ventral translation	Poll-Withers	Hard	131.8	2.2	127.4	136.1	43.5	1.4	40.8	46.2
		Soft	143.2	2.2	138.8	147.6	44.5	1.4	41.8	47.2
	Withers-T13	Hard	36.9	0.8	35.4	38.5	21.1	0.6	19.8	22.3
		Soft	38.5	0.8	37.0	40.0	22.2	0.6	21.0	23.5
	T13-T18	Hard	24.6	0.5	23.7	25.5	9.1	0.3	8.5	9.6
		Soft	26.1	0.5	25.2	27.0	9.6	0.3	9.0	10.1
	T18-L3	Hard	18.2	0.4	17.5	18.9	8.1	0.2	7.6	8.5
		Soft	19.1	0.4	18.4	19.8	8.6	0.2	8.2	9.1
	L3-Sacrum	Hard	21.5	0.5	20.6	22.4	11.5	0.3	11.0	12.1
		Soft	21.8	0.5	20.9	22.7	12.2	0.3	11.7	12.8
	Sacrum-Tail	Hard	28.6	0.5	27.5	29.7	15.7	0.5	14.6	16.8
		Soft	28.1	0.5	27.0	29.1	17.7	0.5	16.6	18.7

### Differential rotational movements

#### Discipline

In walk, discipline had a significant effect on all differential rotational movements at T13-T18 (*p* < 0.039); axial rotation was reduced by 3.1°, flexion–extension decreased by 1.1° and lateral bending was reduced by 0.5° in the load pullers compared to the non-load pullers. In addition, axial rotation was 1.8° lower at Withers-T13 and flexion–extension was 1.6° lower at Poll-Withers in the load pullers.

In trot, the impact of discipline was also observed most in the T13-T18 region, where axial rotation, flexion–extension and lateral bending decreased by 2.3°, 1.7° and 0.6°, respectively, for the load pullers. As per the walk exercise, axial rotation was also lower at Withers-T13 (by 2.1°) and flexion–extension was also lower at Poll-Withers (by 1.6°) in the load pullers; and, additionally, lateral bending was lower at the T18-L3 region (by 0.5°) (Table 3 and Fig. 2).

#### Surface

Surface effects on differential rotational movements in the thoraco-lumbo-sacral spine were common in both walk and trot. The soft surface tended to increase differential rotational movement in both gaits, with increases ranging from 0.2–2.9º. In walk, axial rotation was higher on the soft surface at all anatomical regions, by 0.3–1.2°, except for Poll-Withers and T13-T18, which were unaffected by surface. Flexion–extension was also significantly higher on the soft surface at all sites (by 0.1–1.3°), except for T13-T18, where it was reduced by 0.1°. The amount of lateral bending increased on the soft surface by 2.9° at Poll-Withers. Smaller increases in lateral bending on the soft surface were also observed at L3-Sacrum and Sacrum-Tail (0.4°, in each case) but there was a decrease of 0.1° at T18-L3, and no influence of surface at Withers-T13 and T13-T18.

In trot, the response to surface in terms of axial rotation was consistent with walk, with an increase on the soft surface at all sites (0.7–2.7°) except for Poll-Withers and T13-T18, which were unaffected by surface. With the exception of T13-T18, flexion–extension was also impacted at most anatomical sites, with increases of 0.2–1.1° being observed on the soft surface relative to the hard surface. Lateral bending also increased at all anatomical sites, although the increase was not significant at Withers-T13. As per the walk exercise, the greatest difference between surfaces was observed at Poll-Withers (2.9°), whereas differences of 0.3–0.5° were found elsewhere (Table 4 and Fig. 3).

### Differential translational movements

#### Discipline

Load pullers showed a general pattern of decreased differential translational movements, compared to non-load pullers. In walk, differential cranio-caudal displacement at L3-sacrum was affected by discipline, with 2.2 mm lower displacement in the load pullers compared to the non-load pullers. Medio-lateral displacement was generally not impacted greatly by discipline, although at Withers-T13 and T13-T18 there was 10.3 mm and 3.8 mm less displacement, respectively, in the load pullers compared to the non-load pullers. Dorso-ventral displacement was reduced at all sites in the load pullers, apart from Sacrum-Tail. The most noticeable difference was at Poll-Withers, where dorso-ventral displacement was 15.0 mm less in the load pullers compared to the non-load pullers. Elsewhere, at the other anatomical sites, a 3.1–5.6 mm reduction was found in the load pullers, compared to the non-load pullers.

In trot, differential cranio-caudal displacement was also reduced at L3-sacrum in the load pullers compared to the non-load pullers (by 1.2 mm) but there was 12.8 mm greater differential cranio-caudal displacement at Poll-Withers in the load pullers, and this difference was four times more pronounced compared to at walk (3.2 mm). Differential medio-lateral displacement was 1.5–7.6 mm less at Withers-T13, T13-T18 and T18-L3 in the load pullers compared to the non-load pullers. Differential dorso-ventral displacement at the anatomical sites was less affected by discipline at trot compared to walk, although it did decrease at Poll-Withers and L3-Sacrum, by 7.4 mm and 1.5 mm respectively. It is worth noting that the magnitudes of differential translational movements for both disciplines were reduced in trot compared to walk (Table 3, Fig. 4).

#### Surface

Surface had a strong impact on the differential translational movements at both walk and trot. In walk, differential cranio-caudal displacement increased by 16.3 mm and 2.5 mm at Poll-Withers and Withers-T13, respectively, on the soft surface compared to the hard surface. In contrast, there were small decreases in differential cranio-caudal displacement at T13-T18 and T18-L3 (0.7 mm and 0.3 mm, respectively) on the soft surface. Differential cranio-caudal displacement at L3-Sacrum and Sacrum-Tail were unaffected by the surface type. Differential medio-lateral displacement was found to be significantly greater at Poll-Withers, Withers-T13 and T13-T18, by 1.4–10 mm, and again the offset was greatest around the Poll-Withers. There was a small differential medio-lateral displacement decrease, of 2.2 mm, on the soft surface compared to the hard surface at Sacrum-Tail. Apart from L3-Sacrum and Sacrum-Tail, all anatomical sites showed greater differential dorso-ventral displacement on the soft surface, with the greatest difference between the surfaces being observed at Poll-Withers (11.4 mm).

In trot, differential cranio-caudal displacement was reduced by 2.2 mm at Poll-Withers but increased by 0.6–1.1 mm on the soft surface compared to the hard surface across the other anatomical sites, except for T13-T18, where there was no impact of surface on differential cranio-caudal displacement. Differential medio-lateral displacement increased by 0.5–4.3 mm across all anatomical sites on the soft surface, compared to the hard surface, except for Poll-Withers, where there was no significant effect of surface. Differential dorso-ventral displacement was also modestly affected by surface, with increases of 0.5–2 mm on the soft surface, across anatomical sites. Differential dorso-ventral displacement at Poll-Withers was also unaffected by surface (Tables 2, 4, and Fig. 5).

### Covariates

The covariates appearing in the models were evaluated at the following values: 1223–1224 ms for stride time in walk, and 727 ms for stride time in trot, and 183–185 months for age. The covariates had weak relationships with the differential rotational and translational movements. These relationships are explored and presented in the Supplementary Material with loess curves (Figures S5–S16).

## Discussion

Suppleness and an effective range of motion in the back, are significant contributors to good health and performance success in horses [23, 62, 63]. However, back movement in the horse is poorly understood compared to, for example, lameness and movement asymmetry. There is a need to objectively quantify back movement in horses, to evaluate, without bias, their response to work demands, management, and treatment. Furthermore, the development of a reference framework using healthy individuals is essential for identifying changes in back range of motion that may signify injury or disease. This study is constructive towards developing such a framework, by tackling first and foremost how normal back movement presents in a sub-group of horses from the military (or police) forces. This work established that surface conditions commonly influence the differential rotational and translational movements in many regions of the thoraco-lumbo-sacral spine during walking and trotting exercises. In contrast, horse discipline (load versus non-load pulling) had a more modest effect on thoraco-lumbo-sacral movement. Stride time and age had a limited impact on differential rotational and translational movements.

IMU technology has previously been used to quantity equine back motion practically and non-invasively [13, 14, 16, 29–32, 38, 39, 49] and it has been validated against motion capture methods for quantifying equine locomotion. IMU–derived measurements demonstrate strong agreement with motion capture for assessing both translational and rotational back movements in horses [60, 61]. However, when considering the results presented, it is important to be aware that the IMUs are affected by skin movement and are therefore a proxy for the underlying bone motion. Although bone-fixated sensors measure bone motions more precisely than skin-mounted sensors [10], the ethical implications of such invasive techniques limit their use. Also, some larger differential displacement differences observed at the Poll-Withers and Sacrum-Tail may be attributed to unsteady head movements and tail lifting.

Published data on equine back movement has demonstrated that the magnitude of the amplitude differences between anatomical sites that are spaced-out (several centimetres) along the back are in a measurable range for both medio-lateral and dorso-ventral movements [17, 25, 61]. These amplitude differences exceed several millimetres, and a 3 mm threshold has typically been used in movement asymmetry assessments as a minimum value for detection. Our data indicate that there are differential displacement differences across anatomical regions that are  > 3 mm, consistent with previous work and within detectable ranges. The overall magnitude of the differential rotational movements along the spine reported here are also comparable to previous work [31, 32]. Furthermore, our work was able to identify significant differences between the differential rotational and translational movements in many regions of the thoraco-lumbo-sacral spine during walking and trotting exercises that were related to surface conditions and horse discipline (load versus non-load pulling) effects. These were in the mm-range for differential translational movements or several degrees for differential rotational movements.

In all instances where differential rotational movements were affected by discipline, this presented as a reduced rotation in the load pullers, and the mid-thoracic to caudal regions were most affected. For example, there was a reduction in differential rotational movements in the load-pullers at T13-T18, which was significant, in all cases, at both gaits. Interpreting the consequences of reduced range of motion is challenging. It is commonly accepted that ‘stabilisation’ is positive (or desirable), and ‘stiffness’ is negative (or undesirable), yet both terms describe a reduction in range of motion. It may be more useful to consider spinal motion as a continuum between (pathological) hypomobility and hypermobility, with ‘healthy’ movement falling somewhere between these two extremes. The reduction in rotational ranges of movement in the load pullers is not thought to be influenced by age, as load pullers tended to represent more of the younger horses in our sample set (Figures S11–S16). However, the fact that they represent a younger cohort might suggest that the load-pulling horses are being retired sooner, possibly due to health issues. The reduction in their rotational ranges of movement may signify a potential ‘stiffness’, which might place these horses more vulnerable to back pain. However, the clinical relevance of the significant differences in back movements between the two discipline groups requires further investigation. It is a limitation of our study that we do not know for certain that the horses in our study were sound, and free from back pathologies, and whether they stayed sound over the following months after data collection. It is worth acknowledging that reducing overall flexion and extension movement at or near the thoraco-lumbar junction has been clinically associated with back pain [62], so our findings may suggest that long-term monitoring and potential intervention may be worthwhile in these individuals. The increasing opportunities to apply inertial sensing technologies should facilitate such long-term monitoring of back mobility in horses [16, 61].

There was reduced cranio-caudal displacement at L3-Sacrum in load pullers compared to non-load pullers (particularly at trot) and this area of the spine plays a strong role in transmitting the horizontal thrust force required to pull the guns. Minimising movement in any other direction beyond the direction of the pulling force should facilitate stability and efficiency when pulling loads, hence the reduction in differential cranio-caudal translation in this anatomical region could imply greater stability. In contrast to load pulling horses, the joints of non-load pulling (ridden-only) horses may be exposed to more frequent movements in the vertical plane, resulting in a higher magnitude of dorso-ventral movements. This may be related to the additional mass of the rider, as the lumbar spine has been shown to extend in horses trotting on a treadmill with the addition of a saddle and lead weight (75 kg) [6]. A high magnitude of differential dorso-ventral movements is perhaps unsurprising in the horses at the DATR, which regularly participate in jumping exercise and make up a high proportion of our non-load pulling horses (Table 1). Jumping horses must flex their joint angles (hip, stifle, tibiotarsal, fetlock, and coffin joints) to achieve high vertical impulse to lift the total vertical load, which includes both horse and rider [51]. Furthermore, the repetitive demand to move joints in the thoraco-lumbo-sacral region through greater angular distances over a jump in a set time (and hence greater angular accelerations), may result in long-term alterations to the spinal kinematics in the dorsoventral plane. This is functionally relevant because excessive displacement differences between anatomical sites might be associated with strain on joints, potential injury, or the development of pathologies. It has also been observed that load pulling horses have reduced stance times in both forelimbs and hindlimbs [24], in contrast to horses under mounted load, which instead increase their stance durations [2, 56]; this may be due to differences in vertical versus horizontal ground reaction forces and limb loading associated with the different disciplines [2], and these repetitive differences in kinematics may further impact musculoskeletal health over the long term. It is a limitation of this study that not all horses were participating in equal work at the time of data collection, and there were differences in current activity status (active versus retired). There was also an imbalance in the number of individuals that were retired from load pulling and non-load pulling disciplines (Table 1). Nonetheless, we were still able to identify significant differences in the back movements between the load pulling and non-load pulling horses. Also, when the currently active horses were considered alone, the data revealed trends that were broadly consistent with those assessed using the full dataset (Figures S1–S4), suggesting key differences related to discipline and surface conditions were not masked by individual variability related to active or retired status.

Thoraco-lumbo-sacral movement differences between hard and soft surface were common, and where present, always reflected an increase in differential rotational movement magnitude and most commonly an increase in differential translational movement magnitudes on the soft surface. Soft surfaces are generally more inconsistent and uneven compared to hard surfaces. This may lead to poorer stabilisation of the limbs and upper trunk during locomotion, and an associated higher variability in rotational range of motion across the anatomical sites of the thoraco-lumbo-sacral spine. This is consistent with the observation that soft surfaces induce higher levels of average muscle activity and peak activity in most of the multifidi locations compared to hard surfaces [59]. The increase in dorso-ventral displacement on the soft surface in our study is logical, as soft surfaces result in greater hoof sink during initial ground contact, and greater elastic deformation of the surface [54] and greater return of energy to the hoof to aid propulsion. Gallop data from racehorses have previously indicated that dorso-ventral translational displacement of a horse’s centre of mass is greater on soft artificial surfaces, compared to firmer turf surfaces [19]. As per the gallop data, some of the increases in cranio-caudal movement on the soft surface here could be related to surface differences in grip; there may be higher vertical sink and more limited horizonal translation of the hooves on a softer surface [20] and this could conceivably lead to higher horizonal forces and greater cranio-caudal translation. However, an increase in differential cranio-caudal movements was not universal along the spine, and some sites experienced a decrease in cranio-caudal movement on the soft surface. The reductions in cranio-caudal movement in these instances could be related to the behaviour of the nuchal ligament, which plays a role in energy storage and cervical extension [65]. Previous work has identified that, during both walking and trotting, there is decreased cervicothoracic extension and decreased mid-cervical and low-cervical flexion on a soft surface compared to on a hard surface (asphalt). On softer surfaces, there may be decreased storage of elastic energy and conversion into kinetic energy, leading to a decreased cervicothoracic extension [5] and potentially also decreased cranio-caudal movement. This requires further investigation. In addition, it is worth noting that the ‘soft’ and ‘hard’ surface conditions evaluated in our study varied between data collection sites (Table S1) and were subject to change due to weather conditions and/or repetitive use; for example, the soft surfaces were typically freshly harrowed at the start of the day but became increasingly disturbed over a data collection session.

It is a limitation of our study that the walk and trot speeds varied between trials and that the horses differed in age and conformation. Stride time and speed can be correlated [21, 41, 64] and previous work has indicated that translational displacements along the spine increase with increasing walking speed [22], and translational and rotational movements can increase with walking speed during water treadmill work [39]. In addition, physical properties of muscle change over time [28] and there may be increased muscle atrophy with age [18], which could lead to reduced stabilisation by core musculature and thereby alter rotational and translational movements. However, our data suggest that the model covariates’ (stride time and age) were poorly correlated with the thoraco-lumbo-sacral differential rotational and translational movements (Supplementary Material, Figures S5–S16). This may suggest that stride time (within gait) and age are poor predictors of (normal) back movement in the horse, and that the application of IMU technology for exploring back health in pathological cases may not be impacted greatly by these potential contraindications. Nonetheless, because the model corrections for the covariates revealed some significant differences (Table 2) between disciplines and surfaces that were not always obvious in the raw data boxplots (Figs. 2, 3, 4 and 5), there may be a more nuanced effect of stride time and/or age on the data (Figures S5–S16).

An important next step will be to quantify how abnormal back movement presents in the horse and the interrelation between movement asymmetry and differential translational and rotational back movements. The IMU technology used in this study is transferrable for evaluating conditions of the thoraco-lumbo-sacral spine that impact horses, including, for example, muscle/ligament strains and osteoarthritis of the articular processes. By developing our understanding of how discipline, surface and gait affect movement dynamics in normal horses, we have provided a foundation to explore shifts towards pathological hypomobility or hypermobility that may lead to pain, movement compensation and potential lameness. The prevalence of lameness issues in ridden military horses, which are involved in ceremonial duties and preparation activities (including road work at walk and trot, hacking, schooling and jumping), has been identified to be 25% over one year, with several horses experiencing multiple lameness episodes within this interval [48]. Moving forwards, the development of large databases with many clinical cases spanning a range of pathological conditions will help to better understand the role of different pathologies on range of back movement, which is essential for establishing optimal care and management, high welfare standards and improved public perception of horse use in the equestrian industry. This work should pave the way for future objective dynamic assessments tracking horses’ response to medication, surgery, and rehabilitation.

## Conclusion

This project employed practical and precise non-invasive methods for quantifying differential rotational and translational movements along the equine spine, by capitalising on technological advances in inertial sensing technology. The role of working discipline during straight-line walk and trot exercises was evaluated on hard and soft surfaces, accounting for the potential confounding effects of age and stride time. Horses whose working discipline is primarily pulling loads had reductions in the magnitude of differential rotational movements compared to non-load pulling horses; differential translational movements were also typically reduced in the load pullers, except for cranio-caudal movement at the Poll-Withers during trot. Trotting exercise also tended to be associated with reduced differential ranges of rotational and translational movement compared to walking exercise. In contrast, a soft surface was generally associated with increases in the magnitude of differential rotational and translational movements, compared to a hard surface. Further work is required to understand the significance of these movement differences from a health and performance perspective, and their potential influence on interpretations during clinical gait assessments. Our future work will also explore these data in the context of horse movement asymmetry, back shape, and objective surface properties. In addition, collecting data from clinical cases with known pathologies will provide further context for understanding the impact of diseases on equine back dynamics.

## Supplementary Information

Supplementary Material 1.

## Data Availability

Model data supporting the findings of this study are available within the Main Manuscript. Raw data are available here: https://doi.org/10.6084/m9.figshare.32393460

## Abbreviations

BCS: Body Condition Score
CPU: Central Processing Unit
DATR: Defence Animal Training Regiment
GB: Gigabytes
GPS: Global Positioning System
IBM: International Business Machines
IMU: Inertial Measurement Unit
KWPN: Koninklijk Warmbloed Paardenstamboek Nederland (Dutch Warmblood)
L3: Third lumbar vertebra
LMM: Linear Mixed Model
RAM: Random Access Memory
ROM: Range of Motion
SPSS: Statistical Package for the Social Sciences
T8: Thoracic vertebra 8
T13: Thoracic vertebra 13
T18: Thoracic vertebra 18
UK: United Kingdom
URN: Unique Reference Number
VST: Vienna Surface Tester
