# Supplementary material for: Differential rotational and translational movements along the thoraco-lumbo-sacral spine of load and non-load pulling military and police horses walking and trotting on hard and soft surfaces
Source: BMC Vet Res. 2026 Sep 8;22:559. doi: 10.1186/s12917-026-05804-1 (PMC13613595; doi:10.1186/s12917-026-05804-1)
Supplement: Supplementary file 1 [file 12917_2026_5804_MOESM1_ESM.docx]

**Supplementary Material**

**Differential rotational and translational movements along the thoraco-lumbo-sacral spine of load and non-load pulling military and police horses walking and trotting on hard and soft surfaces**

**Horan, K.^a^***, Warner, S.^a^, Day, P.^a^, Housby-Skeggs, N.^b^, Lane-Ley, E.^c^, Telfer, H.^d^ & Berner, D.^e^

^a^The Royal Veterinary College, Hawkshead Lane, Hatfield, Hertfordshire, AL9 7TA, UK.

^b^The Horse Trust, Slad Lane, Princes Risborough, Buckinghamshire, HP27 0PP, UK.

^c^The King’s Troop Royal Horse Artillery, King George VI Lines, Repository Road, Woolwich, Greater London, SE18 4BB, UK.

^d^Defence Animal Training Regiment, Remount Barracks, Asfordby Road, Melton Mowbray, Leicestershire, LE13 0HX, UK.

*Corresponding author: khoran@rvc.ac.uk

This supplement has five key components, as follows:

1. A summary table characterising the physical properties of the 10 surfaces (five hard and five soft) that were used in this study (Table S1).
2. A visual comparison of the full horse dataset (n=107), evaluated in the main manuscript, to the active only horses (n=66); retired horses were excluded from the plots presented (Figures S1–S4). Overall differential range of motion magnitudes and differences between disciplines and surfaces show broadly consistent patterns for both horse groups at walk and trot.
3. A summary table showing the differences with regards discipline and surface effects between linear mixed models that did and did not include age as a covariate (Table S2). There were 144 models (72 for walk and 72 for trot) and hence 288 comparisons in total. Out of the 288 comparisons, there were 18 instances where the exclusion of age as a covariate generated a different outcome (i.e., where the models did not agree with regards to the effect of discipline and surface effects). Two instances of disagreement were in relation to surface effects and 16 were in relation to discipline effects. There were 10 cases where the model excluding age as a covariate identified a significant difference between disciplines where the original model did not. However, it is possible that some of these differences have been caused by a Type 1 error.
4. A visual summary of the relationships between the differential range of motion parameters and stride time (included as a model covariate) using loess curves (Figures S5–S10).
5. A visual summary of the relationships between the differential range of motion parameters and age (included as a model covariate) using loess curves (Figures S11–S16).

**Table S1 Physical properties of the five hard surfaces (A–E) and the five soft surfaces (F–J) used in this study**. Impact acceleration, penetration depth, energy restitution and stiffness were quantified on the soft surfaces at impact velocities of 2 m/s (v=2) and 4 m/s (v=4) using linear regression analyses.

| **Date**  **(DD/MM/YYYY)** | **Location** | **Hard surface** | | | | **Soft surface** | | | | | | | | | | | | | | | | | |
| --- | --- | --- | --- | --- | --- | --- | --- | --- | --- | --- | --- | --- | --- | --- | --- | --- | --- | --- | --- | --- | --- | --- | --- |
|  |  | **Surface**  **ID** | **Average torque**  **±2 SD (Nm)** | | | **Surface**  **ID** | **Impact**  **acceleration (G)** | | **Penetration**  **depth (mm)** | | **Energy**  **restitution (%)** | | **Stiffness (kN/m)** | | **Average moisture**  **±2 SD (%)** | | | **Temperature**  **±2 SD (degrees)** | | | **Average torque**  **±2 SD (Nm)** | | |
|  |  |  |  |  |  |  | **v=2** | **v=4** | **v=2** | **v=4** | **v=2** | **v=4** | **v=2** | **v=4** |  |  |  |  |  |  |  |  |  |
| 14/02/2023 | DATR | A | 19.1 | ± | 4.7 | F | 50.1 | 128.4 | 10.3 | 16.0 | 2.9 | 3.6 | 345.3 | 627.1 | 0.0 | ± | 0.0 | 8.1 | ± | 0.7 | 21.0 | ± | 5.4 |
| 21/02/2023 | DATR | A | 15.4 | ± | 6.2 | F | 46.0 | 115.4 | 11.7 | 17.7 | 2.6 | 3.2 | 296.3 | 503.1 | 0.0 | ± | 0.0 | 9.8 | ± | 0.5 | 23.0 | ± | 8.7 |
| 07/03/2023 | The Horse Trust - Speen | B | 27.9 | ± | 12.0 | G | 27.6 | 66.6 | 17.1 | 25.7 | 1.0 | 0.5 | 110.5 | 168.2 | 7.5 | ± | 1.2 | 4.8 | ± | 0.4 | 12.4 | ± | 2.5 |
| 14/03/2023 | The Horse Trust - Speen | B | 21.6 | ± | 5.6 | G | 28.8 | 66.8 | 17.0 | 25.7 | 1.2 | 0.9 | 115.4 | 176.3 | 7.6 | ± | 3.7 | 7.7 | ± | 1.0 | 13.4 | ± | 5.3 |
| 22/03/2023 | DATR | A | 22.7 | ± | 8.6 | F | 44.1 | 122.0 | 12.0 | 17.8 | 2.9 | 3.6 | 275.6 | 587.2 | 0.0 | ± | 0.0 | 11.6 | ± | 1.1 | 19.8 | ± | 5.0 |
| 23/03/2023 | DATR | A | 19.9 | ± | 5.6 | H | 37.7 | 97.4 | 15.4 | 21.4 | 2.9 | 3.4 | 200.8 | 369.5 | 3.8 | ± | 5.0 | 11.1 | ± | 1.3 | 13.5 | ± | 2.3 |
| 23/03/2023 | DATR | A |  |  |  | H | 38.6 | 90.2 | 15.7 | 25.1 | 2.9 | 2.9 | 210.4 | 328.2 |  |  |  |  |  |  |  |  |  |
| 11/04/2023 | The Horse Trust - Speen | B | 24.7 | ± | 3.9 | G | 29.1 | 79.9 | 17.5 | 22.4 | 1.2 | 1.0 | 123.6 | 248.0 | 7.6 | ± | 0.9 | 10.4 | ± | 1.1 | 10.5 | ± | 3.8 |
| 18/04/2023 | The Horse Trust - Speen | B | 21.7 | ± | 8.3 | G | 26.1 | 72.8 | 16.4 | 23.3 | 0.9 | 0.6 | 103.5 | 206.0 |  | ± |  | 12.2 | ± | 1.1 | 11.2 | ± | 4.4 |
| 24/04/2023 | The Horse Trust - Speen | B | 22.4 | ± | 6.6 | G | 26.3 | 64.5 | 17.5 | 26.2 | 1.2 | 0.5 | 98.6 | 171.7 | 7.3 | ± | 1.8 | 12.3 | ± | 0.7 | 10.6 | ± | 1.4 |
| 04/07/2023 | The Horse Trust - Speen | B | 16.0 | ± | 3.4 | G | 13.6 | 28.7 | 29.1 | 45.8 | 0.4 | 0.2 | 28.3 | 35.5 | 4.3 | ± | 5.7 | 17.4 | ± | 0.7 | 8.9 | ± | 1.8 |
| 04/07/2023 | The Horse Trust - Speen | B |  |  |  | G | 17.5 | 49.2 | 23.9 | 32.4 | 0.9 | 0.4 | 49.7 | 95.9 |  |  |  |  |  |  |  |  |  |
| 22/07/2449 | The Horse Trust - Langley | C | 15.7 | ± | 7.2 | I |  |  |  |  |  |  |  |  | 2.6 | ± | 5.1 | 22.5 | ± | 1.3 | 11.3 | ± | 3.9 |
| 24/08/2023 | The Horse Trust - Langley | C | 14.8 | ± | 6.2 | I | 23.8 | 65.5 | 19.9 | 28.4 | 1.9 | 1.4 | 87.2 | 163.2 | 0.2 | ± | 0.8 | 17.7 | ± | 0.8 | 11.9 | ± | 1.9 |
| 24/08/2023 | The Horse Trust - Speen | D | 27.1 | ± | 7.1 |  |  |  |  |  |  |  |  |  |  |  |  |  |  |  |  |  |  |
| 20/10/2023 | The Kings Troop | E | 9.3 | ± | 1.5 | J | 38.4 | 84.5 | 14.1 | 25.3 | 2.2 | 2.1 | 201.8 | 290.6 | 4.6 | ± | 4.6 | 15.6 | ± | 0.8 | 11.1 | ± | 3.0 |
| 23/10/2023 | The Kings Troop | E | 10.4 | ± | 4.1 | J | 31.6 | 87.9 | 16.9 | 23.0 | 1.6 | 1.8 | 140.9 | 301.6 | 4.1 | ± | 6.0 | 13.1 | ± | 0.6 | 13.4 | ± | 3.0 |
| 25/10/2023 | The Kings Troop | E | 11.4 | ± | 5.5 | J | 31.2 | 79.3 | 18.0 | 25.5 | 1.6 | 1.5 | 139.8 | 241.9 | 6.6 | ± | 4.5 | 12.8 | ± | 0.6 | 13.0 | ± | 5.3 |
| 27/11/2023 | The Kings Troop | E | 13.3 | ± | 5.1 | J | 37.1 | 100.1 | 15.8 | 21.6 | 2.2 | 2.5 | 203.6 | 402.2 | 4.2 | ± |  | 7.2 | ± | 1.0 | 12.3 | ± | 3.3 |

**Figure S1 Influence of discipline on differential rotational back movements in military horses. A)** Full horse dataset (n=107), which was evaluated in statistical analyses and presented in the main manuscript. Anatomical regions found to be significantly affected by discipline in the linear mixed models are marked (*). **B)** Data from currently active only horses (n=68; no retired horses) are provided for visual comparison to the full horse dataset; statistical analyses were not performed on this reduced dataset.

**Figure S2 Influence of surface on differential rotational back movements in military horses. A)** Full horse dataset (n=107), which was evaluated in statistical analyses and presented in the main manuscript. Anatomical regions found to be significantly affected by surface in the linear mixed models are marked (*). **B)** Data from currently active only horses (n=68; no retired horses) are provided for visual comparison to the full horse dataset; statistical analyses were not performed on this reduced dataset.

**Figure S3 Influence of discipline on differential translational back movements in military horses. A)** Full horse dataset (n=107), which was evaluated in statistical analyses and presented in the main manuscript. Anatomical regions found to be significantly affected by discipline in the linear mixed models are marked (*). **B)** Data from currently active only horses (n=68; no retired horses) are provided for visual comparison to the full horse dataset; statistical analyses were not performed on this reduced dataset.

**Figure S4 Influence of surface on differential translational back movements in military horses. A)** Full horse dataset (n=107), which was evaluated in statistical analyses and presented in the main manuscript. Anatomical regions found to be significantly affected by surface in the linear mixed models are marked (*). **B)** Data from currently active only horses (n=68; no retired horses) are provided for visual comparison to the full horse dataset; statistical analyses were not performed on this reduced dataset.

**Table S2 Comparison of outcomes from linear mixed models with and without the age covariate.** Yellow indicates that there was a significant difference between the disciplines (load pulling versus non-loadpulling) or surfaces (hard versus soft), and red indicates that there was no significant difference between the disciplines or surfaces. Darker shading highlights disagreements between the linear mixed models (model “A” with age covariate, versus model “A’ ” without age covariate): there were 18 instances where the models did not agree. Table footnotes outline the significance values and estimated marginal means in the 10 instances where a significant difference was only identified in models A’; these are also marked in the table by dark blue boxes numbered 1 to 10. The results from models “A” are presented in the main manuscript.

| **Anatomical Location** | **Factor** | **Differential axial rotation (roll)** | | | | **Differential flexion and extension (pitch)** | | | | **Differential lateral bending (yaw)** | | | | **Differential cranio-caudal translation** | | | | **Differential medio-lateral translation** | | | | **Differential dorso-ventral translation** | | | |
| --- | --- | --- | --- | --- | --- | --- | --- | --- | --- | --- | --- | --- | --- | --- | --- | --- | --- | --- | --- | --- | --- | --- | --- | --- | --- |
|  |  | **Walk** | | **Trot** | | **Walk** | | **Trot** | | **Walk** | | **Trot** | | **Walk** | | **Trot** | | **Walk** | | **Trot** | | **Walk** | | **Trot** | |
|  |  | **A** | **A’** | **A** | **A’** | **A** | **A’** | **A** | **A’** | **A** | **A’** | **A** | **A’** | **A** | **A’** | **A** | **A’** | **A** | **A’** | **A** | **A’** | **A** | **A’** | **A** | **A’** |
| Poll-Withers | Discipline |  |  |  |  |  |  |  |  |  |  |  |  |  |  |  |  |  |  |  |  |  |  |  |  |
|  | Surface |  |  |  |  |  |  |  |  |  |  |  |  |  |  |  |  |  |  |  |  |  |  |  |  |
| Withers-T13 | Discipline |  |  |  |  |  |  |  |  |  |  |  |  |  |  |  |  |  |  |  |  |  |  |  |  |
|  | Surface |  |  |  |  |  |  |  |  |  |  |  |  |  |  |  |  |  |  |  |  |  |  |  |  |
| T13-T18 | Discipline |  |  |  |  |  |  |  |  |  |  |  |  |  | **7** |  |  |  |  |  |  |  |  |  | **9** |
|  | Surface |  |  |  |  |  |  |  |  |  |  |  |  |  |  |  |  |  |  |  |  |  |  |  |  |
| T18-L3 | Discipline |  |  |  | **2** |  | **4** |  |  |  |  |  |  |  |  |  |  |  |  |  |  |  |  |  | **10** |
|  | Surface |  |  |  |  |  |  |  |  |  |  |  |  |  |  |  |  |  |  |  |  |  |  |  |  |
| L3-Sacrum | Discipline |  | **1** |  | **3** |  |  |  |  |  |  |  |  |  |  |  |  |  |  |  | **8** |  |  |  |  |
|  | Surface |  |  |  |  |  |  |  |  |  |  |  |  |  |  |  |  |  |  |  |  |  |  |  |  |
| Sacrum-Tail | Discipline |  |  |  |  |  | **5** |  |  |  |  |  | **6** |  |  |  |  |  |  |  |  |  |  |  |  |
|  | Surface |  |  |  |  |  |  |  |  |  |  |  |  |  |  |  |  |  |  |  |  |  |  |  |  |

1. Model A’ discipline effect on axial rotation at L3-Sacrum in walk (p=0.004): EMM for load pullers = 13.9 degrees, EMM for non-load pullers = 16.8 degrees.

2. Model A’ discipline effect on axial rotation at T18-L3 in trot (p=0.002): EMM for load pullers = 12.4 degrees, EMM for non-load pullers = 14.7 degrees.

3. Model A’ discipline effect on axial rotation at L3-Sacrum in trot (p=0.012): EMM for load pullers = 10.6 degrees, EMM for non-load pullers = 12.7 degrees.

4. Model A’ discipline effect on flexion and extension at T18-L3 in walk (p=0.010): EMM for load pullers = 2.6 degrees, EMM for non-load pullers = 3.1 degrees.

5. Model A’ discipline effect on flexion and extension at Sacrum-Tail in walk (p=0.038): EMM for load pullers = 4.1 degrees, EMM for non-load pullers = 4.9 degrees.

6. Model A’ discipline effect on lateral bending at Sacrum-Tail in trot (p=0.039): EMM for load pullers = 4.2 mm, EMM for non-load pullers = 5.1 mm.

7. Model A’ discipline effect on cranio-caudal translation at T13-T18 in walk (p=0.024): EMM for load pullers = 7.8 mm, EMM for non-load pullers = 9.2 mm.

8. Model A’ discipline effect on medio-lateral translation at L3-Sacrum in trot (p=0.045): EMM for load pullers = 20.3 mm, EMM for non-load pullers = 22.6 mm.

9. Model A’ discipline effect on dorsoventral translation at T13-T18 in trot (p=0.007): EMM for load pullers = 8.6 mm, EMM for non-load pullers = 10.1 mm.

10. Model A’ discipline effect on dorsoventral translation at T18-L3 in trot (p=0.039): EMM for load pullers = 7.8 mm, EMM for non-load pullers = 8.8 mm.

**Figure S5 Influence of stride time on differential axial rotation back movements in military horses.**

**Figure S6 Influence of stride time on differential flexion and extension back movements in military horses.**

**Figure S7 Influence of stride time on differential lateral-bending back movements in military horses.**

**Figure S8 Influence of stride time on differential cranio-caudal back movements in military horses.**

**Figure S9 Influence of stride time on differential medio-lateral back movements in military horses.**

**Figure S10 Influence of stride time on differential dorso-ventral back movements in military horses.**

**Figure S11 Influence of age on differential axial rotation back movements in military horses.****Figure S12 Influence of age on differential flexion and extension back movements in military horses.****Figure S13 Influence of age on differential lateral bending back movements in military horses.****Figure S14 Influence of age on differential cranio-caudal back movements in military horses.****Figure S15 Influence of age on differential medio-lateral back movements in military horses.****Figure S16 Influence of age on differential dorso-ventral back movements in military horses.**
